# A Transfer Model Based on Supervised Multi-Layer Dictionary Learning for Brain Tumor MRI Image Recognition

**DOI:** 10.3389/fnins.2021.687496

**Published:** 2021-05-28

**Authors:** Yi Gu, Kang Li

**Affiliations:** School of Artificial Intelligence and Computer Science, Jiangnan University, Wuxi, China

**Keywords:** brain tumor MRI image, supervised learning, transfer learning, Laplacian regularization, multi-layer dictionary learning

## Abstract

Artificial intelligence (AI) is an effective technology for automatic brain tumor MRI image recognition. The training of an AI model requires a large number of labeled data, but medical data needs to be labeled by professional clinicians, which makes data collection complex and expensive. Moreover, a traditional AI model requires that the training data and test data must follow the independent and identically distributed. To solve this problem, we propose a transfer model based on supervised multi-layer dictionary learning (TSMDL) for brain tumor MRI image recognition in this paper. With the help of the knowledge learned from related domains, the goal of this model is to solve the task of transfer learning where the target domain has only a small number of labeled samples. Based on the framework of multi-layer dictionary learning, the proposed model learns the common shared dictionary of source and target domains in each layer to explore the intrinsic connections and shared information between different domains. At the same time, by making full use of the label information of samples, the Laplacian regularization term is introduced to make the dictionary coding of similar samples as close as possible and the dictionary coding of different class samples as different as possible. The recognition experiments on brain MRI image datasets REMBRANDT and Figshare show that the model performs better than competitive state of-the-art methods.

## Introduction

Brain tumor is a common neurological disease. As a high incidence disease, its incidence rate has reached 1.34 per 100,000 in China, and over 200,000 patients diagnosed with primary or metastatic brain tumors in the United States every year. Among the incidence of systemic tumors, brain tumors are second only to those of the stomach, uterus, breast, and esophagus, accounting for approximately 2% of systemic tumors and the proportion of deaths has exceeded 2% (Sun et al., 2019; Sung et al., 2021). According to surveys, the incidence rate of brain tumors is the highest among children, and the highest incidence is 20–50-year-old young adults. Among childhood malignancies, brain tumors are the second most common, after leukemia. Brain tumors not only cause physical and mental suffering to patients, but also place a heavy financial burden on their families. As a standard technique for non-invasive brain tumor diagnosis, magnetic resonance imaging (MRI) is an essential component of medical diagnosis and treatment. It uses magnetic resonance phenomena to obtain electromagnetic signals from the brain, so as to reconstruct brain information and provide a validated anatomical image of the brain. MRI can increase the diagnostic ability of medical diagnosticians. The wide application of MRI mainly benefits from the following characteristics (Amin et al., 2017; Bahadure et al., 2017): (1) no bony artifacts, good soft tissue resolution and clear visualization of soft tissue structures; (2) ability to image multiple aspects and multiple parameters, facilitating the acquisition of diagnostic information as a means of determining the various characteristics of the lesion; (3) no radiological damage and no ionizing radiation damage; (4) different profiles can be selected by adjusting the magnetic field, resulting in a three-dimensional image with different angles, which facilitates the identification of the lesion site; (5) has a flow-space effect and does not require an external contrast agent, allowing direct visualization of the vascular structure and facilitating the observation of the relationship between the vessel and the lesion. However, it is time consuming for radiologists to interpret the large number of MRI images and detect early brain tumors. These medical images need to be analyzed by doctors one by one, and the condition should be determined according to their experience.

Artificial intelligence (AI) technology, especially in particular medical image processing, is an effective way to address this challenge (Zeng et al., 2018; Sajjad et al., 2019; Mittal et al., 2019; Ge et al., 2020; Hua et al., 2021). In the process of brain disease diagnosis, firstly, the image features are extracted, and then the extracted image features are classified to complete the image classification and recognition. For example, Ismael and Abdel-Qader (2018) used Gabor filter and discrete wavelet transform to extract statistical features for brain tumor classification. Then this method used the tumor segmented as input and multi-layer perceptron (MLP) as the classifier. Liu et al. (2012) proposed a multi-level classification method for meningiomas. According to the type and growth rate of tumors, meningiomas are divided into three levels. In the classification step, the authors used a multiple logistic regression model. Mallick et al. (2019) proposed a brain MRI image classification method based on deep neural networks. Using encoding and decoding techniques, this method mainly used an automatic autoencoder to extract and classify brain images. To assist radiologists in MRI classification, Sachdeva et al. (2016) proposed a semi-automated classification method with multiple stages. To detect tumor regions, the first stage was the outline system detection of the content-based tumor regions, which can be manually indicated by the physician, called segmented regions of interest (SROI). Then, 71 texture and intensity features were extracted from the SROI regions, and the features were optimized by genetic algorithm. In the classification stage, support vector machine (SVM) and artificial neural network were used. Nikam and Shinde (2013) proposed a brain MRI image classification method based on distance learning. Firstly, the images were preprocessed, and many techniques such as gray transformation, median filtering, and high pass filtering were used to remove the noise of MRI brain image. The threshold segmentation method was used to segment the MRI brain image. Then the features are extracted by correlation, entropy, contrast, homogeneity, and energy. Finally, a Euclidean distance classifier was used for classification. Ghassemi et al. (2020) proposed a CNN model for multi-class brain tumor classification. Firstly, the method was pre-trained as a discriminator in generative adversarial network to extract image features. Second, the softmax classifier was used to distinguish the three kinds of tumors. This model consists of six layers, which can be used together with various data augmentation techniques. Kiranmayee et al. (2016) proposed a brain MRI classification method using a SVM. In the data processing stage, a median adaptive filter was used to remove noise, and then the watershed method, fuzzy clustering method, and threshold method were used to segment MRI brain image. The kernel SVM was used as the classifier.

The dictionary learning method is widely used to solve various problems of computer vision and image analysis (Gu et al., 2020; Ni et al., 2020). Dictionary learning aims to find a suitable dictionary for the input data and transform it into a sparse representation, so as to mine the useful features of the data, simplify the learning task and reduce the complexity of the model. A kernel sparse representation was developed in Chen et al. (2017). It contained three key steps for multi-label brain tumor segmentation: component analysis-split for dictionary learning initialization, kernel dictionary learning and kernel sparse coding, and graph-cut method for image segmentation. A system combining an adaptive type-2 fuzzy system and dictionary learning was proposed in Ghasemi et al. (2020), in which the sparse coding step and dictionary learning step were executed alternately, and the fuzzy membership functions in the type-2 fuzzy system were used to represent model uncertainty and improve sparse representation. A learning method combining discriminate sub-dictionary and projective dictionary pair learning was developed for classifying proton magnetic resonance spectroscopy of brain gliomas tumor (Adebileje et al., 2017).

AI mainly uses intelligent methods to extract brain image features, which requires a large number of labeled data sets to understand the potential connections in the data. But in the field of medicine, because of the confidentiality and professionalism of patient information, medical data need to be marked by professional clinicians, and data collection is complex and expensive. Lack of labeled trainable data is one of the bottlenecks that restrict the development of medical image analysis. In addition, traditional AI methods require training data and test data to be independent and identically distributed. Transfer learning relaxes this restriction on training data and test data (Ni et al., 2018b; Jiang et al., 2020; Jiang et al., 2021). It can apply the knowledge or patterns learned from a related domain (source domain) to another target domain, and utilize the information shared by source domain samples and target domains, then finally build a model to adapt to the target domain.

To solve this problem, this paper focuses on solving the distribution differences between source and target domains. Through the feature mapping of source and the target domain samples, the source domain knowledge can be transferred to target domain learning. Because dictionary learning can exploit the essential characteristics of the data, this paper uses Multi-layer dictionary learning (MDL) in transfer learning to exploit the shared knowledge between source and target domains. MDL first obtains the dictionary and sparse features of the first layer on the original samples, then obtains the dictionary and sparse features of the second layer based on the obtained sparse features of the first layer, and learns the dictionary and sparse features in turn to finally obtain the deep dictionary and sparse features. Finally, the new test data can be encoded by the multi-layer dictionary and the final classification results can be obtained. According to the difference of domain and task, transfer learning is divided into feature transfer, sample transfer and parameter transfer. In this paper, the target and source domain are images, and the task is to train the image, extract features, and realize the classification of different types of images, so this paper belongs to the parameter transfer mode. The advantages of this algorithm are as follows: (1) based on multi-layer learning, multi-layer dictionaries are obtained, and the discriminability of sparse representation coefficients can be enhanced in layer by layer dictionary learning; (2) through multi-layer shared dictionary learning, the sample reconstructions of source and target domains are constrained layer by layer, so as to minimize the error of sample reconstruction both in source and target domains; (3) by utilizing the label information, Laplacian regularization term is introduced, and the sparse coding of samples in the same class is as close as possible, while the sparse coding of samples in different classes is as different as possible. At the same time, in the last layer of the proposed model, the classification error term is introduced in the last layer of MDL to improve the discriminative performance of the model; (4) The recognition experiments on brain MRI image datasets REMBRANDT (Clark et al., 2013) and Figshare (Cheng et al., 2016) show that the proposed model performs satisfactory classification performance in terms of accuracy, precision, F1-score, and recall.

The rest of the paper is organized as follows: the related work is introduced in section “Backgrounds.” The proposed method is given in section “Proposed Method”, and experiments are performed in section “Experiment.” Finally, conclusion and future work are summarized in section “Conclusion.”

## Backgrounds

### Dictionary Learning

Dictionary learning methods can basically be divided into unsupervised dictionary learning and supervised dictionary learning. The unsupervised dictionary learning does not make use of sample label information. The supervised dictionary learning makes use of sample label information and pays more attention to the discriminative ability of sparse representation coefficients.

KSVD (Jiang et al., 2013) is a famous supervised dictionary learning algorithm. KSVD introduces the classification error of a linear classifier into the objective function, while learning the representation and classification ability of the dictionary. The objective function of K-SVD is

(1) $$\begin{array}{c} {<D,W,Z\geq m\,i\,n|\,\left|\text{X}\text{ -}\text{DZ}\right||}_{2}^{2}+\gamma \,{||\text{H}\text{ -}\text{WZ}||}_{2}^{2}+\beta \,{||W||}_{2}^{2}\\ s.t.{||Z||}_{0}\leq T \end{array}$$

where **Z** is the sparse representation coefficient, **W** is the parameter of the linear classifier, **H** is the label vector of the training data. To solve Eq. (1), the first two of these terms are combined and Eq. (1) is rewritten as

(2) $$\begin{array}{c} {<D,W,Z\geq \min|\,\left|\left(\begin{array}{c} \text{X}\\ \sqrt{\gamma }\,\text{H} \end{array}\right)-\left(\begin{array}{c} \text{D}\\ \sqrt{\gamma }\,\text{W} \end{array}\right)\,\text{Z}\right||}_{2}^{2}+\beta \,{||W||}_{2}^{2}\\ s.t.{||Z||}_{0}\leq T \end{array}$$

Eq. (2) can be solved by using an iterative strategy. When **W** is fixed, the problem of <**D,Z**> represents the same formulation as K-SVD, and it can therefore be solved using the K-SVD. When **D** and **Z** are fixed, Eq. (2) is a simple linear problem that can be solved by linear methods.

### Multi-Layer Dictionary Learning

**FIGURE 1:**
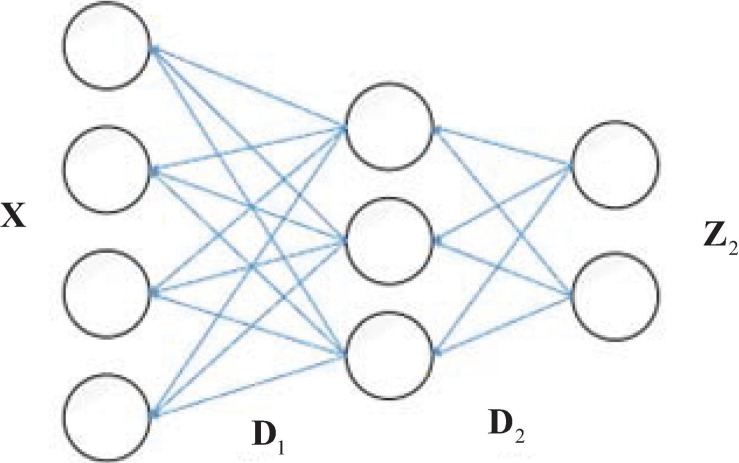
The schematic diagram of two-layer dictionary learning.

With the development of deep learning, researchers have found that the deeper the structure of a neural network, the better and more accurate the representation. MDL (also known as deep dictionary learning) refers to the idea of deep learning, and applies “deep structure” to layer-by-layer dictionary learning (Song et al., 2019; Gu et al., 2020). The dictionary and sparse representation obtained by the traditional single-layer dictionary learning method are shallow, which is not conducive to the task of recognition and classification when the data dimension is too high or the number of samples is too large. Singhal et al. (2017) proposed a deep dictionary learning model, which used the idea of deep learning to learn the multi-level dictionary and the deep features of the original samples. As an example, the two-layer dictionary learning is illustrated in Figure 1. **D**_1_ and **D**_2_ are dictionaries learned in the first and second layer. **Z**_2_ is the sparse coefficient learned in the second layer. The sample **X** can be represented as *X* = **D**_1_**Z**_1_ = **D**_1_**D**_2_**Z**_2_, where the sparse coding learning in the first layer **Z**_1_ = **D**_2_**Z**_2_. Specifically, the first layer is solved as a single layer of dictionary learning to obtain feature **Z**_1_ on dictionary **D**_1_, and **Z**_1_ is then used as input to the second layer, which is also solved as a single layer of dictionary learning to obtain feature **Z**_2_. The new test data can be encoded by the learned **D**_1_ and **D**_2_. In this way, after completing the *L*-layer dictionary learning, the final dictionary and sparse representations are obtained as **D**_*L*_ and **Z**_*L*_. In this case, the sample **X** can be represented as

(3) $$\text{X}={\text{D}}_{1}\,\left({\text{D}}_{2}\,\left(\ldots \,\left({\text{D}}_{L}\,{\text{Z}}_{L}\right)\right)\right)$$

Then the dictionaries in *L*-layers and the sparse coding can be solved by

(4) $$\min_{D_{1},D_{2},\ldots ,D_{L},Z_{L}}{||{\text{X-D}}_{1}\,\left({\text{D}}_{2}\,\left(\ldots \,\left({\text{D}}_{L}\,{\text{Z}}_{L}\right)\right)\right)||}_{F}^{2}+{||{\text{Z}}_{L}||}_{1}$$

## Proposed Method

### Objective Function

We assume that there is a corresponding association between source and target domains in transfer learning. From this point, based on the framework of MDL, we try to learn the common shared dictionary between source and target domains to exploit the shared knowledge among different related domains. At the same time, by making full use of the label information of the samples, the classification error term is introduced in the last layer of the multi-layer dictionary, which makes the sparse representation of the target domain more discriminative. According to this idea, we propose a transfer model based on supervised multi-layer dictionary learning (TSMDL), and its objective function is

(5) $$\begin{array}{l} \underset{Z,w,b}{\min}{\left\|X_{s}-D_{1}Z_{1}^{s}\right\|}_{F}^{2}+{\left\|X_{t}-D_{1}Z_{1}^{t}\right\|}_{F}^{2}+\alpha_{1}\sum_{i,j}Q_{1,i,j}^{C,s}{\left\|z_{1,i}^{s}-z_{1,j}^{s}\right\|}_{F}^{2}-\beta_{1}\sum_{i,j}Q_{1,i,j}^{M,s}{\left\|z_{1,i}^{s}-z_{1,j}^{s}\right\|}_{F}^{2}\\ \;+\alpha_{1}\sum_{i,j}Q_{1,i,j}^{C,t}{\left\|z_{1,i}^{t}-z_{1,j}^{t}\right\|}_{F}^{2}-\beta_{1}\sum_{i,j}Q_{1,i,j}^{M,t}{\left\|z_{1,i}^{t}-z_{1,j}^{t}\right\|}_{F}^{2}+...\\ \;+{\left\|Z_{L-1}^{s}-D_{L}A_{L}^{s}\right\|}_{F}^{2}+{\left\|Z_{L-1}^{t}-D_{L}A_{L}^{t}\right\|}_{F}^{2}+\alpha_{L}\sum_{i,j}Q_{L,i,j}^{C,s}{\left\|z_{L,i}^{s}-z_{L,j}^{s}\right\|}_{F}^{2}-\beta_{L}\sum_{i,j}Q_{L,i,j}^{M,s}{\left\|z_{L,i}^{s}-z_{L,j}^{s}\right\|}_{F}^{2}\\ \;+\alpha_{L}\sum_{i,j}Q_{L,i,j}^{C,t}{\left\|z_{L,i}^{t}-z_{L,j}^{t}\right\|}_{F}^{2}-\beta_{L}\sum_{i,j}Q_{L,i,j}^{M,t}{\left\|z_{L,i}^{t}-z_{L,j}^{t}\right\|}_{F}^{2}\text{+}\lambda \sum_{c=1}^{C_{s}}f(Z_{L}^{s},y_{c}^{s},w_{c}^{s},b_{c}^{s})\\ \text{ +}\lambda \sum_{c=1}^{C_{t}}f(Z_{L}^{t},y_{c}^{t},w_{c}^{t},b_{c}^{t})\text{+}\lambda_{l}\sum_{l=1}^{L}{\left\|Z_{l}\right\|}_{F}^{2},\\ s.t.\;{\left\|d_{l}^{j}\right\|}_{2}^{2}=1,l=1,2,...,L,\;j=1,2,...,K_{l}\; \end{array}$$

where *K*_*l*_ is the size of dictionary in the *l*th layer. ${\text{Z}}_{l}^{s}$ and ${\text{Z}}_{l}^{s}$ are the spares coding matrixes in the *l*-th layer. ${\text{Q}}_{l}^{C,\left(\cdot \right)}$ and ${\text{Q}}_{l}^{M,\left(\cdot \right)}$ are the matrixes of weights for data samples of the same class and data samples of different classes in the *l*-th layer. $Q_{l,i\,j}^{C,\left(\cdot \right)}$ and $Q_{l,i\,j}^{M,\left(\cdot \right)}$ can be defined as

(6) $$Q_{l,i\,j}^{C,\left(\cdot \right)}=\left\{ \begin{array}{c} \frac{1}{\left|C\right|},\left({\text{z}}_{l,i}^{\left(\cdot \right)},{\text{z}}_{l,j}^{\left(\cdot \right)}\right)\,\text{belonging}\,\mathrm{to}\,\mathrm{the}\,\mathrm{same}\,\mathrm{class}\\ 0,\text{otherwise} \end{array}\right.$$

(7) $$Q_{l,i\,j}^{M,\left(\cdot \right)}=\left\{ \begin{array}{c} \frac{1}{\left|M\right|},\left({\text{z}}_{l,i}^{\left(\cdot \right)},{\text{z}}_{l,j}^{\left(\cdot \right)}\right)\,\text{belonging}\,\mathrm{to}\,\mathrm{different}\,\mathrm{classes}\\ 0,\text{otherwise} \end{array}\right.$$

where (⋅) means *s* or *t*.

We explain the above Eq. (5) as follows:

1. The first two terms ${||{\text{X}}_{s}-{\text{D}}_{1}\,{\text{Z}}_{1}^{s}||}_{F}^{2}$ and ${||{\text{X}}_{t}-{\text{D}}_{1}\,{\text{Z}}_{1}^{t}||}_{F}^{2}$ are the reconstruction error terms of source domain and target domain data in the first layer of the learning framework.
2. The third and fourth terms $\sum_{i,j}{\text{Q}}_{1,i,j}^{C,s}\,{||{\text{z}}_{1,i}^{s}-{\text{z}}_{1,j}^{s}||}_{F}^{2}$ and $\sum_{i,j}{\text{Q}}_{1,i,j}^{M,s}\,{||{\text{z}}_{1,i}^{s}-{\text{z}}_{1,j}^{s}||}_{F}^{2}$ are the Laplacian regularization terms of the source domain in the first layer, which, respectively, constrain the dictionary codes of the same class in the source domain to be as close as possible, and the dictionary codes of different classes to be as different as possible. The fifth and sixth terms $\sum_{i,j}{\text{Q}}_{1,i,j}^{C,t}\,{||{\text{z}}_{1,i}^{t}-{\text{z}}_{1,j}^{t}||}_{F}^{2}$ and $\sum_{i,j}{\text{Q}}_{1,i,j}^{M,t}\,{||{\text{z}}_{1,i}^{t}-{\text{z}}_{1,j}^{t}||}_{F}^{2}$ are the Laplacian regularization terms of the target domain in the first layer. Similarly to the third and fourth terms, their goal is to, respectively, constrain the dictionary codes of the same class in the target domain to be as close as possible, and the dictionary codes of different classes to be as different as possible.
3. Following the generation rules for the first six terms, the corresponding reconstruction error terms and Laplacian regularization terms for the source and target domains are constructed for layers 2 to *L*.
4. $\sum_{c=1}^{C_{s}}f\,\left({\text{Z}}_{L}^{s},{\text{y}}_{c}^{s},{\text{w}}_{c}^{s},{\text{b}}_{c}^{s}\right)$ and $\sum_{c=1}^{C_{t}}f\,\left({\text{Z}}_{L}^{t},{\text{y}}_{c}^{t},{\text{w}}_{c}^{t},{\text{b}}_{c}^{t}\right)$ are classification error terms for the last layer of the source domain and target domain, respectively. Its goal is to improve the discriminative ability of the model. In this study, we use SVM multi-class classifier. The parameters $w_{c}^{\left(\cdot \right)}$ and $b_{c}^{\left(\cdot \right)}$ are hyperplane parameters in the SVM.

Define Laplacian matrix in the same class ${\text{P}}_{l}^{C,\left(\cdot \right)}$ as ${\text{P}}_{l}^{C,\left(\cdot \right)}={\tilde{\text{Q}}}_{l}^{C,\left(\cdot \right)}-{\text{Q}}_{l}^{C,\left(\cdot \right)}$, where ${\tilde{\text{Q}}}_{l,i\,i}^{C,\left(\cdot \right)}=\sum_{j}{\text{Q}}_{l,i\,j}^{C,\left(\cdot \right)}$, Laplacian matrix in the different classes ${\text{P}}_{l}^{M,\left(\cdot \right)}$ as ${\text{P}}_{l}^{M,\left(\cdot \right)}={\tilde{\text{Q}}}_{l}^{M,\left(\cdot \right)}-{\text{Q}}_{l}^{M,\left(\cdot \right)}$, where ${\tilde{\text{Q}}}_{l,i\,i}^{M,\left(\cdot \right)}=\sum_{j}Q_{l,i\,j}^{M,\left(\cdot \right)}$. Let **X** = [**X**_*s*_,**X**_*t*_], **Z** = [**Z**_*s*_,**Z**_*t*_], ${\text{P}}_{l}^{C}=\left[\begin{array}{cc} {\text{P}}_{l}^{C,s} & 0\\ 0 & {\text{P}}_{l}^{C,t} \end{array}\right]$, ${\text{P}}_{l}^{M}=\left[\begin{array}{cc} {\text{P}}_{l}^{M,s} & 0\\ 0 & {\text{P}}_{l}^{M,t} \end{array}\right]$, Eq. (5) can be written as

(8) $$\begin{array}{c} \min_{D_{l},P_{l}^{C},P_{l}^{M},Z_{l},w,b}{\left|{\text{X-D}}_{1}\,{\text{Z}}_{1}\right|}_{F}^{2}+T\,r\,\left({\text{Z}}_{1}\,\left(\alpha_{1}\,{\text{P}}_{1}^{C}+\lambda_{1}\,\text{I}\right)\,{\text{Z}}_{1}^{T}\right)\\ -\beta_{1}\,T\,r\,\left({\text{Z}}_{1}\,{\text{P}}_{1}^{M}\,{\text{Z}}_{1}^{T}\right)+\ldots +\\ {||{\text{Z}}_{L-1}-{\text{D}}_{L}\,{\text{Z}}_{L}||}_{F}^{2}+T\,r\,\left({\text{Z}}_{L}\,\left(\alpha_{L}\,{\text{P}}_{L}^{C}+\lambda_{L}\,\text{I}\right)\,{\text{Z}}_{L}^{T}\right)\\ -\beta_{L}\,T\,r\,\left({\text{Z}}_{L}\,{\text{P}}_{L}^{M}\,{\text{Z}}_{L}^{T}\right)+\lambda \,\sum_{c=1}^{C}f\,\left({\text{Z}}_{L},{\text{y}}_{c},{\text{w}}_{c},{\text{b}}_{c}\right),\\ s.t.{||d_{l}^{j}||}_{2}^{2}=1,l=1,2,\ldots ,L,j=1,2,\ldots ,K_{l}, \end{array}$$

Again, we simplify the function above and obtain that

(9) $$\begin{array}{cc} \min_{D_{l},P_{l}^{C},P_{l}^{M},Z_{l},w,b}||{\text{X-D}}_{1}{\text{Z}}_{1}|{|}_{F}^{2}+Tr({\text{Z}}_{1}\left(\alpha_{1}{\text{P}}_{1}^{C}-\beta_{1}{\text{P}}_{1}^{M}+\lambda_{1}\text{I}\right) & \\ Z_{1}^{T}+\ldots + & \\ ||{\text{Z}}_{L-1}-{\text{D}}_{L}{\text{Z}}_{L}|{|}_{F}^{2}+Tr({\text{Z}}_{L}(\alpha_{L}{\text{P}}_{L}^{C}-\beta_{L}{\text{P}}_{L}^{M} & \\ +\lambda_{L}\text{I}){\text{Z}}_{L}^{T})+\lambda \sum {}_{c=1}{}^{C}f({\text{Z}}_{L},{\text{y}}_{c},{\text{w}}_{c},{\text{b}}_{c}), & \\ s.t.{||d_{l}^{j}||}_{2}^{2}=1,l=1,2,\ldots ,L,j=1,2,\ldots ,K_{l}, & \end{array}$$

### Optimization

We use the alternating optimization approach to solve Eq. (9). The parameters to be solved include **D**_1_, ${\text{P}}_{1}^{C}$, ${\text{P}}_{1}^{M}$, **Z**_1_,…, **D**_*L*_, ${\text{P}}_{L}^{C}$, ${\text{P}}_{L}^{M}$, **Z**_*L*_, ***w*** and *b*. In the following, we divide the solution of these variables into three parts.

a. Update parameters **D**_1_, ${\text{P}}_{1}^{C}$, ${\text{P}}_{1}^{M}$, **Z**_1_,…, **D**_*L*_, ${\text{P}}_{L}^{C}$ and ${\text{P}}_{L}^{M}$

First, we update parameters **D**_1_, ${\text{P}}_{1}^{C}$, ${\text{P}}_{1}^{M}$ and **Z**_1_in the first layer. When fixed the other parameters, the objective function of TSMDL is

(10) $$\begin{array}{c} \min_{D_{1},P_{1}^{C},P_{1}^{M},Z_{1}}||{\text{X-D}}_{1}{\text{Z}}_{1}|{|}_{F}^{2}+Tr({\text{Z}}_{1}\left(\alpha_{1}{\text{P}}_{1}^{C}-\beta_{1}{\text{P}}_{1}^{M}+\lambda_{1}\text{I}\right){\text{Z}}_{1}^{T}\\ +{||{\text{Z}}_{1}-{\text{D}}_{2}\,{\text{Z}}_{2}||}_{F}^{2},\\ s.t.{||d_{1}^{j}||}_{2}^{2}=1 \end{array}$$

Further, the parameters except for **D**_1_ are fixed, the optimization problem can be written as

(11) $$\begin{array}{c} \min_{D_{1},P_{1}^{C},P_{1}^{M},Z_{1}}{||{\text{X-D}}_{1}\,{\text{Z}}_{1}||}_{F}^{2},\\ s.t.{||d_{1}^{j}||}_{2}^{2}=1 \end{array}$$

Following (Boyd et al., 2011), the optimal value of **D**_1_ can be computed by an alternating direction method of multipliers. Then the Laplacian matrixes ${\text{P}}_{1}^{C}$ and ${\text{P}}_{1}^{M}$ can be computed according to Eqs.(6, 7).

The optimal value of **Z**_1_ can be obtained by taking the derivation of Eq.(8) as the following formulation, i.e.,

(12) $${\text{Z}}_{1}={\left({\text{D}}_{1}^{T}\,{\text{D}}_{1}+\alpha_{1}\,{\text{P}}_{1}-\beta_{1}\,{\text{P}}_{1}^{M}+\left(\lambda_{1}+1\right)\,\text{I}\right)}^{-1}\,\left({\text{D}}_{1}^{T}\,\text{X}+{\text{D}}_{2}\,{\text{Z}}_{2}\right)$$

For 2≤*l*≤*L*, when fixing the other parameters, the objective function of **D**_*l*_ is

(13) $$\begin{array}{c} \min_{D_{l}}{||{\text{Z}}_{l-1}-{\text{D}}_{l}\,{\text{Z}}_{l}||}_{F}^{2},\\ s.t.{||d_{2}^{j}||}_{2}^{2}=1,j=1,2,\ldots ,K_{L} \end{array}$$

After obtaining the **D**_*l*_, the optimal value of **Z**_*l*_(2≤*l*≤*L*−1) can be obtained by,

(14) $${\text{Z}}_{l}={\left({\text{D}}_{l}^{T}\,{\text{D}}_{l}+\alpha_{l}\,{\text{P}}_{l}^{C}-\beta_{l}\,{\text{P}}_{l}^{M}+\left(\lambda_{l}+1\right)\,\text{I}\right)}^{-1}\,\left({\text{D}}_{1}^{T}\,\text{X}+{\text{D}}_{l+1}\,{\text{Z}}_{l+1}\right)$$

b. Update parameter **Z**_*L*_:

When the other parameters are fixed, the objective function of TSMDL related to **Z**_*L*_ is

(15) $$\min_{Z_{L}}||{\text{Z}}_{L-1}-{\text{D}}_{L}\,{\text{Z}}_{L}||+T\,r\,\left({\text{Z}}_{L}\,\left(\alpha_{L}\,{\text{P}}_{L}^{C}-\beta_{L}\,{\text{P}}_{L}^{M}+\lambda_{L}\,\text{I}\right)\,{\text{Z}}_{L}^{T}\right)+\lambda \,\sum_{c=1}^{C}f\,\left({\text{Z}}_{L},{\text{y}}_{c},{\text{w}}_{c},{\text{b}}_{c}\right)$$

Let ${\text{z}}_{L}^{i}\left(i=1,2,\ldots ,N\right)$ be the *i*th column of **Z**_*L*_. We rewrite Eq. (15) related to ${\text{z}}_{L}^{i}$ as

(16) $$\min_{Z_{L}}||{\text{z}}_{L-1}^{i}-{\text{D}}_{L}\,{\text{Z}}_{L}||+T\,r\,\left({\text{z}}_{L}^{i}\,\left(\alpha_{L}\,{\text{P}}_{L}^{C}-\beta_{L}\,{\text{P}}_{L}^{M}+\lambda_{L}\,\text{I}\right)\,{\text{z}}_{L}^{i\,T}\right)+\lambda \,\sum_{c=1}^{C}f\,\left({\text{z}}_{L}^{i},{\text{y}}_{c}^{i},{\text{w}}_{c},{\text{b}}_{c}\right)$$

In this study, we use standard L1-SVM for term $f\,\left({\text{z}}_{L}^{i},{\text{y}}_{c}^{i},{\text{w}}_{c},{\text{b}}_{c}\right)$, thus we can set ${\text{y}}_{c}^{i}=1$ if class label ${\text{y}}_{c}^{i}=c$ and otherwise $y_{c}^{i}=-1.$ In this case, the optimal value of ${\text{z}}_{L}^{i}$ can be computed by a least square problem.

c. Update parameters **w** and *b*

When the other parameters are fixed, the objective function of TSMDL related to **w** and *b* is

(17) $$\min_{w_{c},b_{c}}\sum_{c=1}^{C}f\,\left({\text{Z}}_{L},{\text{y}}_{c},{\text{w}}_{c},b_{c}\right),$$

Obviously, Eq. (17) can be solved by various SVM solvers.

**Table:** We show the optimization procedure of TSMDL in algorithm 1.

**Input:** Training data matrix **X**, parameters α_*l*_,β_*l*_ and λ_*l*_, ∀*l*
1: Initialize **D** using K-SVD algorithm on each class, initialize **P** using principal component analysis (PCA) algorithm;
2: **while** not converged **do**
3: Compute **D**_*l*_(1≤*l*≤*L*) by Eq. (11);
4: Compute the Laplacian matrixes ${\text{P}}_{l}^{C}$ and ${\text{P}}_{l}^{M}$(1≤*l*≤*L*) by Eqs. (6, 7);
5: Compute **Z**_*l*_(1≤*l*≤*L*−1) by Eqs. (12–14);
6: Compute ${\text{z}}_{L}^{i}$ by Eq. (16);
7: Compute **w** and *b* by Eq. (17);
8: **end while**
**Output: **D**_1_**,${\text{P}}_{1}^{C}$,${\text{P}}_{1}^{M}$,**Z**_1_,…, **D**_*L*_,${\text{P}}_{L}^{C}$,${\text{P}}_{L}^{M}$,**Z**_*L*_, **w** and *b.*

### Learning a Classifier

We compute **Θ**_**l**_ = ${\left({\text{D}}_{l}^{T}{\text{D}}_{l}+\alpha {\text{P}}_{l}^{C}-\beta {\text{P}}_{l}^{M}+\lambda_{l}\text{I}\right)}^{-1}{\text{D}}_{l}^{T}\left(l=1,2,\ldots ,L\right)$. The test sample **X**_new_, we compute its sparse coding as *z*_new_ = **Θ**_1_…**Θ*L*x**_new_. Finally, we can use the following formulation to predict the class label of **x**_new_

(18) $$\text{y}=\text{arg}\,\underset{c}{\text{max}}{\text{w}}_{c}^{T}\,{\text{z}}_{\text{new}}+b_{c},\forall c$$

## Experiment

### Experiment Settings

**FIGURE 2:**
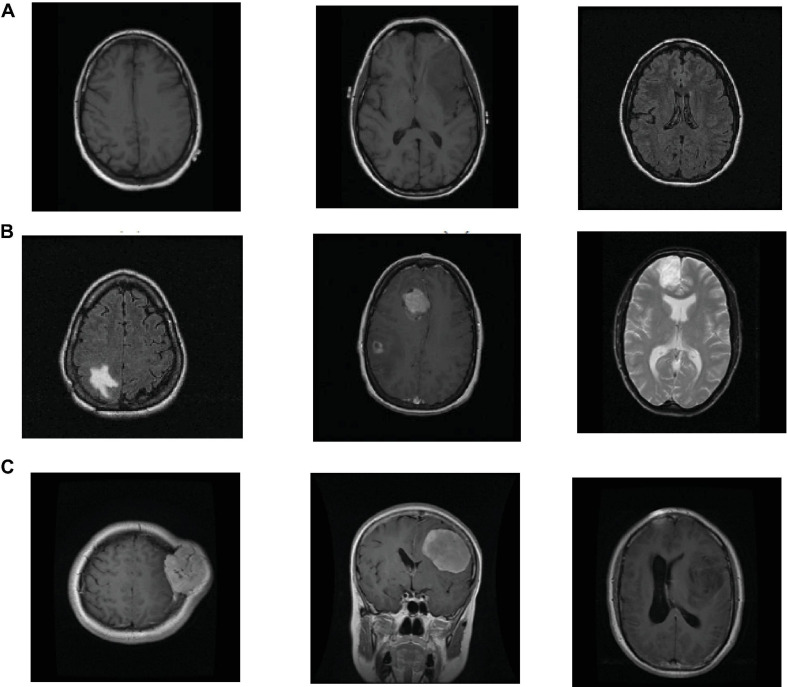
Example samples used in the experiment, **(A)** normal images in the REMBRANDT dataset, **(B)** tumor images in the REMBRANDT dataset, **(C)** tumor images in the Figshare dataset.

The datasets used in the study are taken from (Clark et al., 2013) and Figshare (Cheng et al., 2016) datasets. Figshare dataset is collected from two hospitals in China between 2005 and 2010. It contains a vast number of MRI images from 233 patients with brain tumors, including meningiomas, pituitary and gliomas. All images are digitized at a resolution of 512 × 512 pixels. REMBRANDT dataset contains a vast number of MRI images collected from 130 brain tumor patients. The patients’ ages ranged from 15 to 89 years, with a mean of 47.5 years. The MRI images included astrocytomas from 47 patients; oligodendrogliomas from 21 patients; GBMs from 40 patients and the truth of the tumor in the remaining patients is unknown. All images are digitized at a resolution of 256 × 256 pixels. Some slices in the REMBRANDT dataset, where the tumor lesions are found, are considered normal samples. In the experiment, we design two transfer learning tasks, and show their information in Table 1. Figure 2 shows sample images in the REMBRANDT and Figshare datasets. The main objective of two tasks is to classify the brain MRI images into normal and tumor classes. In task T1, we randomly select 200 normal images and 200 tumor images from the REMBRANDT dataset as source domain, and randomly select 200 normal images from the REMBRANDT dataset and 200 tumor images from Figshare dataset as target domain. There are no duplicate images in source and target domains. We use all images in the source domain and 10% images in the target domain as training data, and use the rest of the images in the target domain as testing data. We use wavelet transform wavelets and gray level co-occurrence matrix (GLCM) method for feature extraction (Mohankumar, 2016). Each image is extracted onto a 540 dimensional vector.

**TABLE 1:** The basic information of transfer learning tasks in the experiment.

Tasks	Source domain		Target domain	
T1	Normal images from the REMBRANDT dataset	Tumors images from the Figshare dataset	Normal images from the REMBRANDT dataset	Tumors images from the REMBRANDT dataset
T2	Normal images from the REMBRANDT dataset	Tumors images from the REMBRANDT dataset	Normal images from the REMBRANDT dataset	Tumors images from the Figshare dataset

In the experiment, we compare our model with LC-KSVD (Jiang et al., 2013), SRC (Wright et al., 2009), CRC (Zhang et al., 2011), HFA (Long et al., 2013), KMA (Tuia and Camps-Valls, 2016), and DDTML (Ni et al., 2018a). Following the authors, all parameters in comparative methods are set in their default settings. The parameters β, λ_*1*_, and λ in TSMDL are set in the grid {0.01, 0.05, 0.1,…,2}. The number of layers is set in {3, 4, 5}, and the TSMDL model is accordingly named as TSMDL-3, TSMDL-4, and TSMDL-5, respectively. The sizes of dictionaries are 500, 450, 400, 350, and 300 corresponding to layer 1 to layer 5, respectively. In order to ensure the stability and effectiveness of the experimental results, for the proposed model and other comparative experimental methods, we run each task 10 times. All the methods are implemented in MATLAB, and the environment that we used in the experiments is a computer with Intel Core i5-3317U 1.70 GHz CPU, 16 GB RAM.

### Experiment Results

**FIGURE 3:**
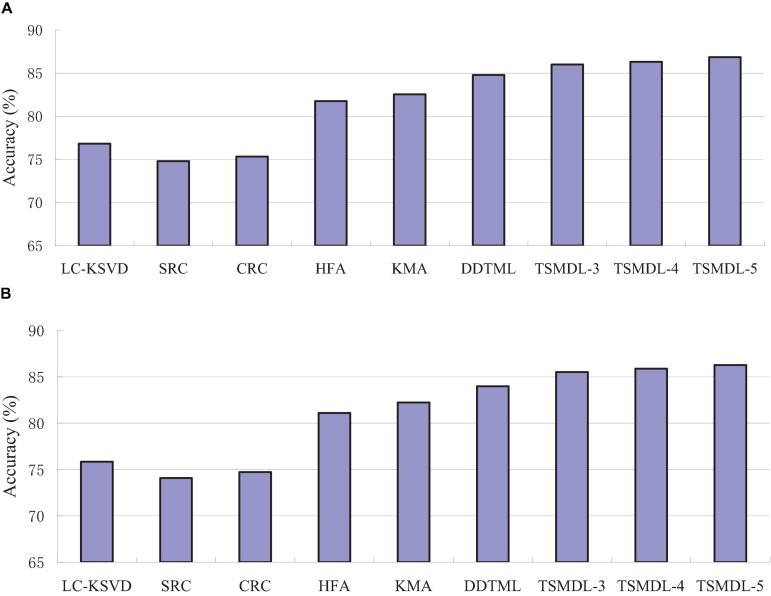
Accuracy comparison results on, **(A)** T1 task, **(B)** T2 task.

**FIGURE 4:**
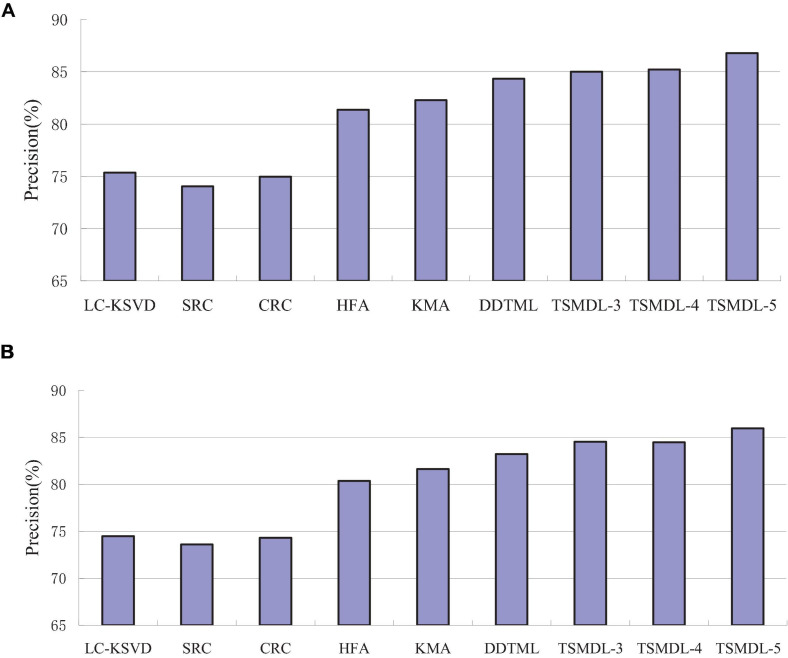
Precision comparison results on tasks, **(A)** T1, **(B)** T2.

**FIGURE 5:**
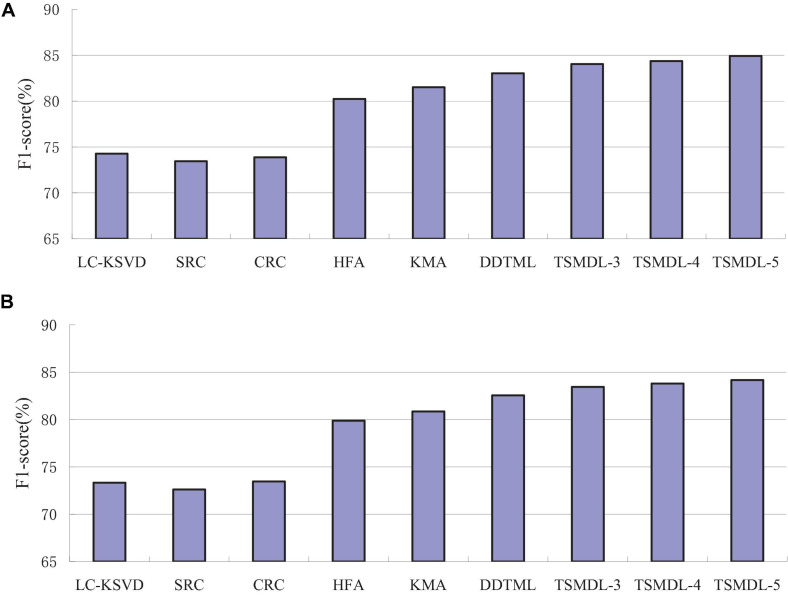
F-Score comparison results on tasks, **(A)** T1, **(B)** T2.

**FIGURE 6:**
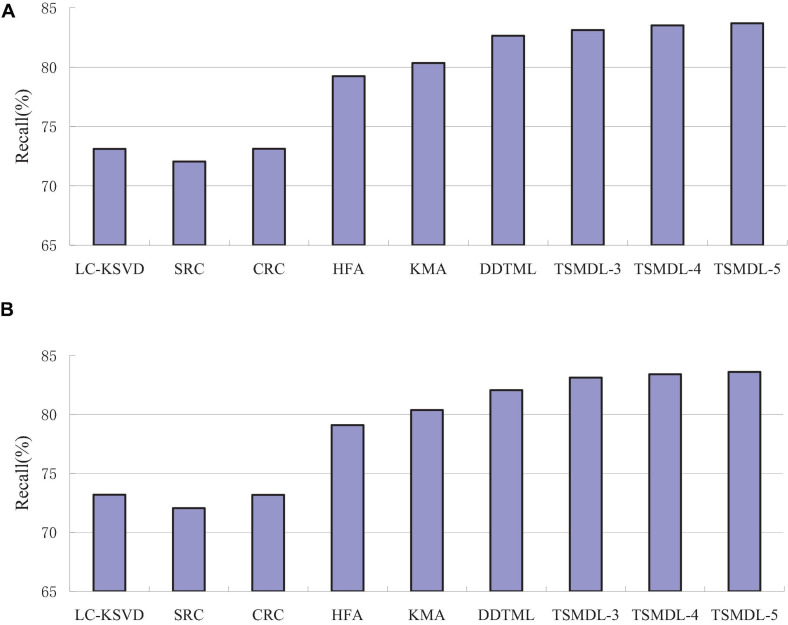
Recall comparison results on tasks, **(A)** T1, **(B)** T2

In this subsection, we present the effect of TSMDL on T1 and T2 tasks. We summarize the performance of all comparative methods in terms of accuracy, precision, F1-score, and recall. The experiment results are shown in Figures 3–6, respectively. According to Figures 3–6, we can draw the following results:

In terms of accuracy, precision, F1-score, and recall, the proposed TSMDL achieves the best results. In addition, the performance of TSMDL-5 is better than TSMDL-3 and TSMDL-4. It is indicated that the multi-layer framework of dictionary learning can exploit the instinct structure of data samples and can build a relationship between source and target domains. Thus, TSMDL is suitable for the application of brain tumor MRI image recognition.

In the experiments, except for the LC-KSVD, SRC, and CRC algorithms, all other algorithms are transfer learning-based classification methods, which show that transfer learning strategy is helpful for brain tumor MRI image classification in the target domain. The classification knowledge in the source domain can be effectively transferred to the target domain to help the target domain achieve better classification results.

The proposed TSMDL in this paper is obviously superior to other transfer learning methods, which shows that multiple layer transfer learning dictionary learning can truly restore the brain MRI images of source and target domains, and reduce the distribution difference between domains. Thus, it can strengthen the domain adoption between source and target domains in the sparse representation space. The reason is that TSMDL is based on MDL; it can learn a more complex and accurate dictionary to represent the original data, and obtain more discriminative representation coefficients. In addition, TSMDL is a supervised learning model, in which the label information can be exploited, so TSMDL can obtain higher discrimination performance.

## Conclusion

With the popularity of MRI equipment, a large number of new MRI brain images emerge, but obtaining labeled data is very time-consuming and expensive. Therefore, the goal of this paper is to use a large number of labeled data from the source domain to learn a classifier with strong generalization ability, and to classify the target domain with only a small number of labeling samples. Therefore, based on the MDL framework, we learn the common dictionary on each layer of the network to minimize the sample reconstruction error of the constrained source domain and target domain. At the same time, the Laplacian regularization term is introduced in each layer of the network to make the sparse coding of similar samples as close as possible, while the sparse coding of different classes of samples is as different as possible. The experimental results on brain MRI image datasets REMBRANDT and Figshare show that our model achieves the state-of-the-art methods. Future works will include studying the effect of using unlabeled samples in the target domain while training, and other relevant problems like large-scale and online adaptation of dictionaries.

## Data Availability Statement

Publicly available datasets were analyzed in this study. This data can be found here: The download URLs of the REMBRANDT and Figshare datasets are, respectively, [https://wiki.cancerimagingarchive.net/display/Public/REMBRANDT] and [https://figshare.com/articles/dataset/brain_tumor_dataset/1512427].

## Author Contributions

YG developed the theoretical framework and model in this work and drafted the manuscript. YG and KL implemented the algorithm and performed experiments and result analysis. Both contributed to the article and approved the submitted version.

## Conflict of Interest

The authors declare that the research was conducted in the absence of any commercial or financial relationships that could be construed as a potential conflict of interest.
